# Severity of mild cognitive impairment in early Parkinson's disease contributes to poorer quality of life

**DOI:** 10.1016/j.parkreldis.2014.07.004

**Published:** 2014-10

**Authors:** Rachael A. Lawson, Alison J. Yarnall, Gordon W. Duncan, Tien K. Khoo, David P. Breen, Roger A. Barker, Daniel Collerton, John-Paul Taylor, David J. Burn

**Affiliations:** a Institute for Ageing and Health, Newcastle University, Newcastle Upon Tyne, UK; b Griffith Health Institute & School of Medicine, Griffith University, Gold Coast, Australia; c John van Geest Centre for Brain Repair, University of Cambridge, Cambridge, UK

**Keywords:** Parkinson's disease, Mild cognitive impairment, Quality of life

## Abstract

**Background:**

Poor quality of life (QoL) is a feature of people with Parkinson's disease (PD) who develop dementia. The relationship between mild cognitive impairment in PD (PD-MCI) and QoL is less clear. To address this, we studied the impact of varying severities of cognitive impairment on QoL in a cohort of non-demented patients with early PD.

**Method:**

Patients with newly diagnosed PD (*n* = 219) and age and sex matched healthy controls (*n* = 99) completed a schedule of neuropsychological tests, in addition to scales assessing QoL (PDQ-39), depression, sleep, neuropsychiatric symptoms and a clinical examination. The Movement Disorder Society criteria were used to define and classify PD-MCI.

**Results:**

Participants with PD-MCI were significantly older than those with normal cognition, had more severe motor symptoms, scored higher for depression and had poorer quality of life. Logistic regression showed that mild cognitive impairment, independent of other factors, was an indicator of poorer QoL. Using cognitive performance 2.0 standard deviations (SD) below normative data as a cut-off to define PD-MCI, there was a significant difference in QoL scores between patients with PD-MCI and those classified as having normal cognition. Subjects with less severe mild cognitive impairment did not exhibit significant differences in QoL.

**Conclusions:**

PD-MCI is a significant, independent factor contributing to poorer QoL in patients with newly diagnosed PD. Those classified with greatest impairment (2.0 SD below normal values) have lower QoL. This has implications for clinical practice and future interventions targeting cognitive impairments.

## Highlights

• Quality of life declines with increased severity of cognitive impairment in PD.
• Mild cognitive impairment in PD (PD-MCI) independently contributes to poorer QoL.
• PD-MCI at 2 standard deviations below controls had the greatest impact on QoL.
• The optimal operational cut-off for PD-MCI may be 2 standard deviations.

## 1 Introduction

Poor quality of life (QoL) and impaired wellbeing are common in Parkinson's disease dementia (PDD) [1], a frequent complication of Parkinson's disease (PD). The point prevalence of PDD is 25–30%, which is six times higher than an aged-matched general population [2]. Cumulatively the prevalence of PDD is estimated to be up to 80% [3]. PDD is associated with increased risk of falls, caregiver burden, nursing home placement and increased mortality [1], [2], [3]. In addition, fractures, urinary incontinence, hallucinations and neuropsychiatric symptoms are also common in PDD and impact on QoL.

The Movement Disorder Society (MDS) Task Force defines mild cognitive impairment in PD (PD-MCI) as performing 1 to 2 standard deviations (SD) below appropriate normative values in neuropsychological tests with no impairments of activities of daily living (ADL) [4]. It is a potential early marker for the development of PDD [5], [6], [7] so may also be associated with poorer QoL [8]. The prevalence of PD-MCI is between 15 and 40% at the time of PD diagnosis [1], [6], [7]. This variability may be due to a lack of consensus in defining PD-MCI, which did not exist until the proposed diagnostic MDS criteria were agreed upon in 2011. A recent review of the literature showed a wide variation in the tests used to diagnose PD-MCI and inconsistencies in the criteria used [9]. The lack of consistent cut-offs leaves much room for variation across studies. A cut-off of below 1.5 SD of normative data of age-matched controls was the most common criterion used.

The relationship between PD-MCI and QoL is unclear; currently only two studies have used the MDS Task Force diagnostic criteria to investigate QoL in PD-MCI [1], [8]. It is vital to understand the contribution MCI and cognitive decline has on PD patients, in addition to the motor and non-motor symptoms, and the extent to which it influences QoL. Understanding the impact of PD-MCI would help guide clinicians as to what pharmacological and non-pharmacological interventions might be particularly effective [10], [11], [12]. We therefore investigated whether the degree of severity of PD-MCI independently influences QoL in patients with newly diagnosed PD. This is also the first study to examine the effects of PD-MCI on QoL in a large cohort of early PD and to explore the impact that different operational cut-offs for diagnosing PD-MCI have on QoL. We hypothesized that those with PD-MCI would have a poorer QoL compared to those with normal cognition.

## 2 Methods

### 2.1 Participants

This study was approved by the Newcastle and North Tyneside Research Ethics Committee and performed according to the Declaration of Helsinki. All participants provided written informed consent.

Participants were recruited from community and outpatient clinics through general practitioners, neurologists, geriatricians and PD nurse specialists in Newcastle upon Tyne, Gateshead and Cambridgeshire as part of the Incidence of Cognitive Impairments in Cohorts with Longitudinal Evaluation-Parkinson's Disease (ICICLE-PD) study. All patients were newly diagnosed with idiopathic PD by a movement disorder specialist and fulfilled Queen's Square Brain Bank criteria [13]. All participants underwent detailed clinical assessment, including carer interviews, to exclude dementia. Participants were excluded if they had significant cognitive impairment at presentation (Mini Mental State Examination (MMSE) < 24) that impaired ADL, met DSM-IV criteria for dementia or a diagnosis of dementia [2]. Age-sex matched healthy controls were recruited through word of mouth and local advertising to provide normative data.

### 2.2 Scales and assessments

Participants and controls completed a schedule of neuropsychological tests. Global cognitive function was assessed using the MMSE [14] and Montreal Cognitive Assessment (MoCA) [15]. Table 1 shows the neuropsychological assessments used. Selective tests from the computerized Cognitive Drug Research (CDR) battery [16] and Cambridge Neuropsychological Test Automated Battery (CANTAB) [17] were used to assess attention, memory and executive function. The phonemic fluency test asked participants to generate as many words as they could in 60 s beginning with the letter F [18]. Similarly, the semantic fluency test asked participants to list as many animals as they could in 90 s [18]. Visuospatial function was evaluated using the pentagon copying item within the MMSE [14] and was graded using a modified 0–2 rating scale [19].

**Table 1:** Neuropsychological tests.

Domain	Test
Attention	CDR:
	Power of attention
	Digit vigilance
Executive function	CANTAB: One touch tower of London (OTS)
	Phonemic fluency
	Semantic fluency
Visuospatial function	Pentagons
Memory	CANTAB:
	Pattern recognition memory (PRM)
	Spatial recognition memory (SRM)
	Paired associate learning (PAL)
Language	MoCA:
	Naming
	Language

CDR = Cognitive Drug Research Battery, CANTAB = Cambridge Neuropsychological Test Automated Battery, MoCA = Montreal Cognitive Assessment.

Consistent with the MDS Task Force criteria [4], participants were classified as having PD-MCI if they scored 1 to 2 SD below the means of appropriate norms (controls) on at least two neuropsychological tests across five cognitive domains: attention, executive function, visuospatial function, memory and language. For data that was not normally distributed and could not be transformed appropriately, percentiles derived from a normal distribution were used to estimate cut-offs 1 SD (16th percentile), 1.5 SD (7th percentile) and 2 SD (2nd percentile), therefore the cut-offs give approximately the correct percentage of people impaired. For example, the pentagon score was assessed as 2 (shape includes 10 angles and clear intersection), 1 (two intersecting figures, one with five angles) or 0 (less acceptable copy); using corresponding percentiles from the control group, participants scoring 1 were classified as having impairment at the 1 SD and 1.5 SD level, and participants scoring 0 were classified as having impairment at the 2 SD level.

Implementation of our schedule of neuropsychological tests preceded the establishment of MDS criteria. However, broadly we were able to meet Level II criteria with our testing, despite having only one test specific for visuospatial impairment. We investigated the differences using 1 SD, 1.5 SD and 2 SD. These three cut-offs have been used in other studies, although 1.5 SD is the most common [8]. Additionally, subjective cognitive decline and functional independence of participants were determined through semi-structured interviews with participants and/or their carers.

Quality of life was measured using the Parkinson's Disease Questionnaire (PDQ-39) [20], which is widely used in PD research and clinics [6]. It includes a 39 item Likert scale covering eight domains: mobility, ADL, emotional wellbeing, stigma, social support, cognition, communication and bodily discomfort. The single index of this scale was used as a global measure of QoL in PD. The single index scores range from 0, (best possible QoL), to 100 (worst possible QoL).

Demographic information, including age, sex and education was collected. Participants also completed the MDS Unified Parkinson's Disease Rating Scale (MDS-UPDRS) Part III [21]. Premorbid intelligence was measured using the National Adult Reading Test (NART) [22]. Depression was assessed using the Geriatric Depression Score (GDS-15) [23]; a cut-off of ≥5 suggested possible depression. Neuropsychiatric symptoms were measured by the Neuropsychiatric Inventory (NPI-D) [24]. Participants were assessed when “on.” Levodopa equivalent dose was calculated for all dopaminergic medications [25].

### 2.3 Statistical analysis

Statistical analyses were performed using SPSS software (Version 19.0; SPSS, Inc., Chicago, IL). Data were examined for normality of distribution with visual histograms and Kolmogorov–Smirnov's test. Comparisons of means between two groups were performed using independent *t*-tests or Mann–Whitney *U* test as appropriate. For more than two group comparison one way ANOVAs or Kruskal–Wallis tests were used as appropriate. Multiple comparisons were corrected using Bonferroni's correction; the cut-off for significance was calculated using *α*/*n* where *α* is the significance level (0.05) and *n* is the number of tests. Logistic regression was used to build a model to predict QoL; data were dichotomized using the median, such that scores below the median were low and scores above the median were high.

## 3 Results

Participants (*n* = 219) were aged between 35 and 87 years (mean of 65.9, SD = 9.7); 63.9% were male (*n* = 140). Mean time since diagnosis was 5.5 months (SD = 5.0), 83% were rated as Hoehn and Yahr stage 1 or 2 and 16% were drug naïve. The ages of the control subjects (*n* = 99) ranged from 48 to 88 years (mean of 67.9, SD = 8.2) and 55% were male (*n* = 54). There was no significant difference between age (*p* = 0.06) and sex (*p* = 0.11) of PD participants and controls. Neither was there a significant difference between PD participants and controls in terms of number of years of education (mean of 13.1 SD = 3.4 and 12.8 SD = 3.6, respectively; *p* = 0.36) and NART scores (mean of 115.8, SD = 8.7 and 114.3, SD = 10.3, respectively; *p* = 0.37).

The clinical characteristics of the cohort according to different levels of PD-MCI are shown in Table 2. PD participants classified as normal cognition (PD-CN) scored within 1 SD of normative data. Three MCI groups were defined: MCI 1 SD which included those with a score of ≥1 SD but <1.5 SD below normative data (23.2%); MCI 1.5 SD which included those with a score ≥1.5 SD but < 2 SD below normative data (21.1%); and MCI 2 SD who scored ≥2 SD below normative data (22.4%). In each group, participants scored below the means of appropriate norms (controls) on at least two neuropsychological tests for that standard deviation.

**Table 2:** Mean and standard deviation of clinical data across four groups.

	Normal cognition (*n* = 75)		MCI 1SD (*n* = 51)		1.5 SD MCI (*n* = 44)		2 SD MCI (*n* = 49)		F/*χ*^2^	
	M	SD	M	SD	M	SD	M	SD		
Age (years)	61.2	10.0	67.3	8.4	68.9	8.8	69.0	8.7	10.6**	
Education (years)	14.4	3.7	13.1	3.3	11.2	2.4	11.4	3.7	42.6**	a,b
NART	118.4	7.2	115.2	10.5	112.1	10.2	108.9	11.6	26.5**	b
PD Duration (months)	5.3	4.9	6.1	4.8	5.8	6.9	4.8	3.3	2.7	
Time since symptom onset (months)	18.7	13.5	26.1	20.6	28.7	37.6	26.7	25.1	6.7	
UPDRS III Total	23.0	9.4	27.2	10.4	31.7	11.9	31.6	13.9	8.6**	
Hoehn and Yahr^a^	2.0	1.0	2.0	0.0	2.0	0.0	2.0	1.0	19.1**	
Levodopa equivalent dose (mg/d)^a^	100.0	220.0	120.0	224.0	130.0	200.0	150.0	200.0	3.2	
MoCA	27.4	1.8	26.1	2.4	24.5	3.2	22.3	3.9	55.5**	a,b,c
MMSE	29.3	0.9	28.8	0.9	28.5	1.3	27.9	1.6	31.7**	b
Phonemic fluency	14.3	4.4	11.8	4.4	9.7	4.0	9.8	4.5	15.0**	
Semantic fluency	25.0	5.8	21.5	5.3	19.1	5.6	16.7	6.8	21.6**	b
GDS-15	2.3	1.9	2.8	2.9	2.9	2.6	4.0	4.0	10.4*	b
NPI Total	6.2	3.9	7.3	4.3	5.6	5.7	7.5	5.4	1.1	
NPI Distress	2.9	9.1	3.5	9.7	3.4	10.1	3.3	10.4	1.0	
PDQ-39	24.3	19.4	26.6	21.9	28.8	21.1	38.2	25.2	11.7**	b

*p < 0.05, **p < 0.01.

Post hoc Bonferroni correction for 3 group comparison at *p* < 0.017, a PD-MCI 1SD vs. PD-MCI 1.5SD; b PD-MCI 1SD vs. PD-MCI 2SD; c PD-MCI 1.5SD vs. PD-MCI 2SD.

NART = National Adult Reading Test, UPDRS III = Movement Disorders Society-Unified Parkinson's Disease Rating Scale Part III, MoCA = Montreal Cognitive Assessment, MMSE = Mini Mental State Examination, GDS-15 = Geriatric Depression Score, NPI = Neuropsychiatric Inventory, PDQ-39 = Parkinson's Disease Questionnaire.

a Figures are median and interquartile range.

**Fig. 1:**
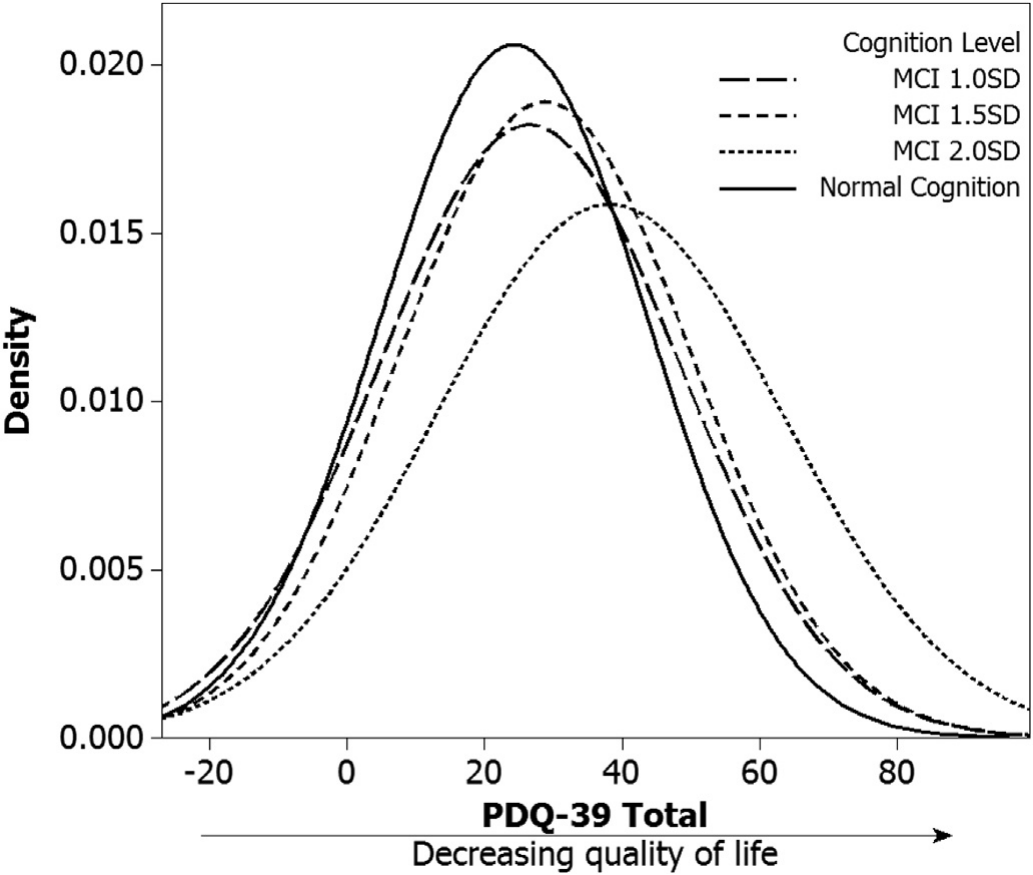
Distribution of quality of life between cognitive groups. Graph of probability density function of PDQ-39 scores for the discreet groups of normal cognition, PD-MCI 1 SD, PD-MCI 1.5 SD and PD-MCI 2 SD.

As a group, participants with PD-MCI (≥1 SD below normative data) were most commonly impaired in executive function (67%), memory (61%) and attention (51%) domains. They were significantly older, had spent fewer years in education and had a lower premorbid IQ (NART) (*p* < 0.01) than PD-CN. They also had a higher MDS-UPDRS III score (*p* < 0.01) and Hoehn and Yahr stage (*p* < 0.01), but there was no significant difference in levodopa equivalent dose. Mean depression scores were significantly higher for PD-MCI group compared to PD-CN, although this is below the suggested cut-off for possible depression (GDS-15 > 5) [23]. Post hoc comparisons between MCI groups (Table 2) showed an overlap in scores for PDQ-39, seen clearly in Fig. 1, highlighting the variation of QoL scores by severity of cognitive impairment. Only the 2 SD group significantly differed from the other groups, hence only more marked cognitive impairment at 2 SDs had a greater impact on QoL. Because of this, we focussed our subsequent analyses using 2 SD as a cut-off for PD-MCI.

### 3.1 Predicting quality of life

Stepwise logistic regression was used to determine predictors of QoL and to remove potential confounders. The final model (Table 3) correctly predicted 72.0% of QoL scores compared to observed values. Goodness of fit tests yielded a Nagelkerke R Square of 0.346. The model shows that higher scores of motor severity, depression and neuropsychiatric symptoms predicted worse QoL (Table 3). The presence of MCI was an independent predictor of poorer QoL. Younger participants were predicted to have lower QoL.

**Table 3:** Logistic regression model predicting quality of life.

Variable	*β*	S.E.	*p* value	Exp(*β*)
Age	1.05	0.39	0.007	2.86
UPDRS III	−0.81	0.38	0.033	0.45
GDS-15	−2.45	0.79	0.002	0.09
MCI	−1.45	0.48	0.003	0.23
NPI Total	−0.95	0.39	0.016	0.39
Constant	3.78	0.91	0.000	43.66

UPDRS III = Movement Disorders Society-Unified Parkinson's Disease Rating Scale Part III, GDS-15 = Geriatric Depression Score, MCI = Mild Cognitive Impairment using 2 stand deviation cut-off, NPI-D = Neuropsychiatric Inventory.

## 4 Discussion

We have shown that MCI at 2 SDs below normative values is an independent, significant predictor of QoL in patients with newly diagnosed PD. QoL declines with increasing cognitive impairment in patients with PD, even when other contributory factors are taken into account. The greatest decline is seen in those who score more than 2 SD below normal on cognitive measures. This cut-off identifies 22% of our cohort as having MCI, which is consistent with other estimates of prevalence [9].

We have shown that increasing cognitive impairment is associated with a number of factors, including depression and PD severity. Therefore the operational definition of MCI used has an influence on the association between MCI and QoL. Thus while QoL scores were statistically different in patients with “milder” PD-MCI, i.e. at 1 SD or 1.5 SD, the effect of cognitive impairment on QoL was strongest if MCI was defined by cognitive performance 2 SD or more below the control mean. Therefore, there is a transition effect at a 2 SD threshold at which cognitive difficulties have a noticeable impact on people's lives. From this perspective we would suggest that 2 SD may be an appropriate operational cut-off for defining MCI in PD.

This transition effect supports the conclusions of Leroi et al. [1], which is one of only two recent studies investigating QoL in PD-MCI using the newly outlined PD-MCI criteria by from MDS Task Force [1], [8]. Leroi et al. assessed QoL and caregiver burden in participants classified as PD-CN, PD-MCI and PDD. As cognitive impairment increased, QoL became more impaired across the three groups, although only PDD was significantly higher [1]. This could be due to the use of Level I criteria of “possible” PD-MCI, which offers less diagnostic certainty than Level II criteria [4]. Our study showed declining QoL with increasing cognitive impairment, with more severe PD-MCI having the greatest decline in QoL. This transition effect could indicate that these participants are likely to develop PDD [5].

Applying the MDS Task Force Level II criteria, Reginold, et al. found changes from premorbid cognition and PD-MCI were associated with reduced QoL in the PDQ-39 sub-scores of stigma, communication and social support, but not in overall QoL (PDQ-39 single index score) [8]. However, cognitive decline was estimated using the Wechsler Test of Adult Reading (WTAR), which may over estimate premorbid intelligence and cognitive decline [22]. Our study, however, found significant differences in the single index PDQ-39 score, and was independently predicted by the presence of PD-MCI.

Attention, memory and executive function, the ability to plan and prioritize, were the domains most commonly affected. Impairment in these domains has previously been directly associated with poorer QoL [26], [27]; inhibiting everyday function and impacting on ADL [12], [26], [28], or resulting in less effective coping strategies [11], [27]. Attentional deficits can impact on instrumental and functional ADL including bathing, eating and leisure activities, such as reading or watching television [12]. Trigg et al. [28] have suggested that awareness of impairment may impact on QoL. Speculatively participants with PD-MCI may have perceived poorer QoL in that they are aware of cognitive changes and the impact they are having on their ADLs, and may draw comparisons to how they are performing now in comparison to their premorbid levels of function [28].

Interestingly, our model showed that younger PD participants were predicted to have poorer QoL. This could reflect that younger participants with PD have higher expectations of QoL than older PD participants or find it harder to adjust, and therefore have perceived poorer QoL [27]. Indeed previous studies have found that older patients with PD are more accepting of disability and impairment as they are perceived as being appropriate to their age [29]. Younger participants may also perceive having PD as “unfair” and are less able to deal with stigma and experience more severe psychosocial consequences [29].

The main strengths of this study are the large cohort of newly diagnosed PD patients and the range of validated instruments used to assess motor and non-motor symptoms, including a detailed schedule of neuropsychological tests. We used the PDQ-39 single index score to measure QoL, which is validated for PD and is widely used [8]. The MDS guidelines state that normative values should be age, education, gender, and culturally appropriate [4]. As demonstrated in the results section, there were no significant differences between controls and PD participants in terms of age, gender, education and premorbid intelligence (NART score). Furthermore, control participants were not spouses or relatives of PD participants to limit potential bias, and were recruited locally through word of mouth and advertising to reflect the community and cultural population. We also examined the scores and cut-offs for cognitive tests using age and education as covariates. However, remodeling our data did not have a significant impact either on PD-MCI classification or on QoL.

There are several limitations. The challenging nature of accurately assessing MCI raises the possibility of falsely identifying some participants as having MCI [4]. However, the use of Level II criteria and the 2 SD as a cut-off for PD-MCI, in addition to semi-structured interviews with participants and/or their carers, increases diagnostic certainly. We used modified MDS criteria since the study design predated the recent PD-MCI guidelines. While the assessments for executive function, attention and memory were suitably covered, we had limited assessments for language and particularly visuospatial function, which included only one domain-specific test, which has implications for classification. However, language has been shown to be relatively preserved in previous studies [9], [30] and impaired ability to copy pentagons has been shown to predict dementia [5]. Furthermore, MoCA scores in patients with PD-MCI at 2 SD (mean score of 22.3) were low in our study and below the cut-off that suggests possible dementia. This mean is nonetheless comparable with MoCA scores for PD-MCI from previous studies [8], and ultimately dementia is not determined by MoCA score alone and all participants underwent rigorous clinical assessment to exclude dementia (including a caregiver interview). Participants were recruited from outpatient clinics and 84% were on antiparkinsonian medication, which reflects current clinical practice.

This cohort will be followed longitudinally as part of a larger incidence study to track the relationship between changes in cognition and variations in QoL. This will establish an accurate diagnosis and ascertain how QoL is affected by declining cognition from PD-MCI to PDD [4]. We have found that cognitive impairment specifically contributes, independently, to poorer QoL even in early PD. More marked degrees of PD-MCI (2 SD below normal values) have a greater impact upon QoL, suggesting a transition effect.

Increased awareness and understanding of the impact of PD-MCI would inform clinicians of which cognition focused interventions, such as cognitive training or cognitive stimulation interventions, are potentially beneficial [9]. Targeting specific cognitive impairments to improve everyday function, ADL and coping strategies may also have a direct positive impact on QoL [26], [27]. Increased understanding would also inform clinicians on disease modifying medication; dopaminergic medication, for example, may have an ameliorating effect on executive function, impacting on QoL [10]. Consequently, studies investigating cognitive or disease modifying interventions should include QoL measures.

## Conflicts of interest

None.

## References

[1] Leroi I., McDonald K., Pantula H., Harbishettar V. (2012). Cognitive impairment in parkinson disease: impact on quality of life, disability, and caregiver burden. J Geriatr Psychiatry Neurol.

[2] Emre M., Aarsland D., Brown R., Burn D.J., Duyckaerts C., Mizuno Y. (2007). Clinical diagnostic criteria for dementia associated with Parkinson's disease. Mov Disorders Official Journal Mov Disord Soc.

[3] Hely M.A., Reid W.G., Adena M.A., Halliday G.M., Morris J.G. (2008). The Sydney multicenter study of Parkinson's disease: the inevitability of dementia at 20 years. Mov Disorders Official Journal Mov Disord Soc.

[4] Litvan I., Goldman J.G., Tröster A.I., Schmand B.A., Weintraub D., Petersen R.C. (2012). Diagnostic criteria for mild cognitive impairment in Parkinson's disease: movement disorder society task force guidelines. Mov Disorders Official Journal Mov Disord Soc.

[5] Williams-Gray C.H., Evans J.R., Goris A., Foltynie T., Ban M., Robbins T.W. (2009). The distinct cognitive syndromes of Parkinson's disease: 5 year follow-up of the CamPaIGN cohort. Brain A Journal Neurology.

[6] Broeders M., de Bie R.M., Velseboer D.C., Speelman J.D., Muslimovic D., Schmand B. (2013). Evolution of mild cognitive impairment in Parkinson disease. Neurology.

[7] Janvin C.C., Larsen J.P., Aarsland D., Hugdahl K. (2006). Subtypes of mild cognitive impairment in Parkinson's disease: progression to dementia. Mov Disorders Official Journal Mov Disord Soc.

[8] Reginold W., Duff-Canning S., Meaney C., Armstrong M.J., Fox S., Rothberg B. (2013). Impact of mild cognitive impairment on health-related quality of life in Parkinson's disease. Dement Geriatr Cogn Disord.

[9] Yarnall A.J., Rochester L., Burn D.J. (2013). Mild cognitive impairment in Parkinson's disease. Age Ageing.

[10] Kehagia A.A., Barker R.A., Robbins T.W. (2010). Neuropsychological and clinical heterogeneity of cognitive impairment and dementia in patients with Parkinson's disease. Lancet Neurol.

[11] Hurt C.S., Landau S., Burn D.J., Hindle J.V., Samuel M., Wilson K. (2012). Cognition, coping, and outcome in Parkinson's disease. Int Psychogeriatr.

[12] Bronnick K., Ehrt U., Emre M., De Deyn P.P., Wesnes K., Tekin S. (2006). Attentional deficits affect activities of daily living in dementia-associated with Parkinson's disease. J Neurol Neurosurg Psychiatr.

[13] Hughes A.J., Daniel S.E., Kilford L., Lees A.J. (1992). Accuracy of clinical diagnosis of idiopathic Parkinson's disease: a clinico-pathological study of 100 cases. J Neurol Neurosurg Psychiatr.

[14] Folstein M.F., Folstein S.E., McHugh P.R. (1975). “Mini-mental state”. A practical method for grading the cognitive state of patients for the clinician. J Psychiatr Res.

[15] Nasreddine Z.S., Phillips N.A., Bédirian V., Charbonneau S., Whitehead V., Collin I. (2005). The montreal cognitive assessment, MoCA: a brief screening tool for mild cognitive impairment. J Am Geriatr Soc.

[16] Nicholl C.G., Lynch S., Kelly C.A., White L., Simpson P.M., Wesnes K.A. (1995). The cognitive drug research computerized assessment system in the evaluation of early dementia-is speed of the essence?. Int J Geriatr Psychiatry.

[17] Robbins T.W., James M., Owen A.M., Sahakian B.J., McInnes L., Rabbitt P. (1994). Cambridge neuropsychological test automated battery (CANTAB): a factor analytic study of a large sample of normal elderly volunteers. Dementia.

[18] Tombaugh T.N., Kozak J., Rees L. (1999). Normative data stratified by age and education for two measures of verbal Fluency: FAS and animal naming. Arch Clin Neuropsychol.

[19] Ala T.A., Hughes L.F., Kyrouac G.A., Ghobrial M.W., Elble R.J. (2001). Pentagon copying is more impaired in dementia with lewy bodies than in Alzheimer's disease. J Neurol Neurosurg Psychiatr.

[20] Jenkinson C., Fitzpatrick R.A.Y., Peto V.I.V., Greenhall R., Hyman N. (1997). The Parkinson's disease questionnaire (PDQ-39): development and validation of a Parkinson's disease summary index score. Age Ageing.

[21] Goetz C.G., Tilley B.C., Shaftman S.R., Stebbins G.T., Fahn S., Martinez-Martin P. (2008). Movement disorder society-sponsored revision of the unified Parkinson's disease rating scale (MDS-UPDRS): scale presentation and clinimetric testing results. Mov Disord Official Journal Mov Disord Soc.

[22] Mathias J.L., Bowden S.C., Barrett-Woodbridge M. (2007). Accuracy of the wechsler test of adult reading (WTAR) and national adult reading test (NART) when estimating IQ in a healthy Australian sample. Aust Psychol.

[23] Yesavage J.A., Sheikh J.I. (1986). Geriatric depression scale (GDS): recent evidence and development of a shorter version. Clin Gerontol.

[24] Cummings J.L., Mega M., Gray K., Rosenberg-Thompson S., Carusi D.A., Gornbein J. (1994). The neuropsychiatric inventory. Neurology.

[25] Tomlinson C.L., Stowe R., Patel S., Rick C., Gray R., Clarke C.E. (2010). Systematic review of levodopa dose equivalency reporting in Parkinson's disease. Mov Disorders Official Journal Mov Disord Soc.

[26] Klepac N., Trkulja V., Relja M., Babic T. (2008). Is quality of life in non-demented Parkinson's disease patients related to cognitive performance? A clinic-based cross-sectional study. Eur J Neurol Official Journal Eur Fed Neurol Soc.

[27] Kudlicka A., Clare L., Hindle J.V. (2014). Quality of life, health status and caregiver burden in Parkinson's disease: relationship to executive functioning. Int J Geriatr Psychiatry.

[28] Trigg R., Watts S., Jones R., Tod A. (2011). Predictors of quality of life ratings from persons with dementia: the role of insight. Int J Geriatr Psychiatry.

[29] Schrag A., Hovris A., Morley D., Quinn N., Jahanshahi M. (2003). Young- versus older-onset Parkinson's disease: impact of disease and psychosocial consequences. Mov Disorders Official Journal Mov Disord Soc.

[30] Pfeiffer H.C., Lokkegaard A., Zoetmulder M., Friberg L., Werdelin L. (2014). Cognitive impairment in early-stage non-demented Parkinson's disease patients. Acta Neurol Scand.

